# Solubilities in Aqueous Solutions of Ammonium Sulfate and Potassium Salts of Malonic, Succinic, or Glutaric Acid

**DOI:** 10.1021/acs.jpca.5c05396

**Published:** 2025-08-19

**Authors:** Keith D. Beyer, Karl T. Taylor, Samuel R. Rendler, Julia L. Roskam

**Affiliations:** Department of Chemistry & Biochemistry, 14750 University of Wisconsin-La Crosse, La Crosse, Wisconsin 54601, United States

## Abstract

The aqueous solubility of the least soluble salt was studied in mixtures of ammonium sulfate and one of the following organic acids in either a 1:1 or 2:1 ratio with KOH:malonic acid, succinic acid, and glutaric acid. Solubility temperatures were determined in solutions of known concentrations using differential scanning calorimetry. The identity of the least soluble solid was determined by a combination of X-ray crystallography and infrared spectroscopy. The identified solids were found to be potassium sulfate (19% of samples), ammonium sulfate (5% of samples), and compounds with varying ratios of NH_4_^+^/K^+^ with SO_4_^2–^ (76% of samples). The organic fraction of solutions appeared to have no impact on the identity of the least soluble solid but did have an impact on that solid’s solubility in the respective solution. In general, the potassium-containing compounds were found to be less soluble than the analogous sodium-containing compounds previously reported.

## Graphical abstract

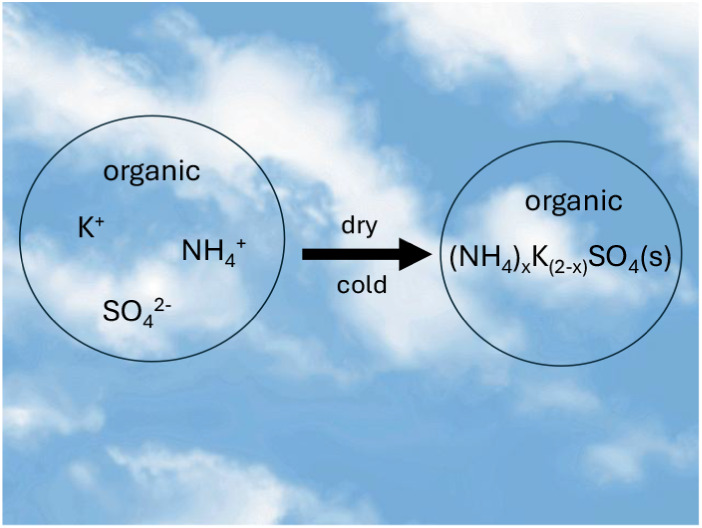

## 1 Introduction

Field measurements have shown that aerosols in the free and upper troposphere (UT) are primarily composed of organic compounds, inorganic constituents, and mineral dust.^1,2^ Among the inorganic species, ammonium and sulfate are the most prevalent.^3^ Within the organic fraction, dicarboxylic acids (DCA) are commonly observed across a range of environments, especially in aerosols that have undergone chemical aging.^4^ Both primary and secondary organic aerosols have been found to contain DCA,^3^ and their concentrations in atmospheric particles are increasing.^5^ Low-carbon-number DCA, such as malonic (C_3_), succinic (C_4_), and glutaric (C_5_) acids, are frequently reported as the most abundant in these aerosols.^5–9^

Mineral dust is also a significant component of atmospheric aerosols, particularly under acidic conditions where chemical aging facilitates the dissolution of dust particles into the aqueous phase.^10,11^ This aqueous-phase chemistry can be reinforced by metals, which may participate in surface reactions or become incorporated into the aerosol interior.^11,12^ Field studies have confirmed the widespread presence of organics, metals, and metal salts in aerosols originating from sources such as sea spray (Na, Mg),^4,13–15^ biomass burning (K),^16,17^ and mineral dusts or meteoritic material (Na, K, Ca, Fe).^2,18–21^ These components are found throughout the atmosphere.^22^

Metal ions in aerosols can replace hydrogen ions in organic acids, forming carboxylate salts—a process supported by both field and laboratory studies.^13,14,23,24^ In the case of a DCA, such as malonic acid, a particle containing dissolved KCl and exposed to cold or dry conditions typical of the UT may undergo the following reaction:

1 $$n\mathrm{KCl}\;\left(\mathrm{aq}\right)+H_{2}C_{3}H_{2}O_{4}\;\left(\mathrm{aq}\right)\to K_{n}H_{\left(2-n\right)}C_{3}H_{2}O_{4}\left(s\right)+n\mathrm{HCl}\;\left(\mathrm{aq},g\right)$$

Here, *n* represents either one or two, depending on how far this reaction proceeds to completion. In this scenario, the malonate ion would remain in the aerosol as a solid salt (low vapor pressure), while the resulting HCl may evaporate (high vapor pressure), as has been observed in lab studies,^13,24^ and making the reaction irreversible. Thus, precipitation of the organic salt may occur under UT-relevant conditions.^23^

Although the solubilities of several potassium oxalate salts have been studied,^25^ the effects of ammonium sulfate (AS) on the solubility of these salts remain unexplored. Furthermore, given the complexity of these multicomponent systems, it is possible that additional salts—beyond the potassium salts of DCA and AS—could form. This was observed to be the case in studies of sodium salts of DCA (malonic,^26^ succinic, and glutaric^27^) in the presence of AS. Additionally, compounds of the formula (NH_4_)_*x*_K_(2–*x*)_SO_4_ have been known to form from aqueous solutions of K_2_SO_4_ and AS as a continuous solid solution.^28–30^ Solubilities of these compounds with various compositions are found in the compilation of Linke.^31^ Thus, the present study was undertaken to determine if organic salts or inorganic salts would be the least soluble in solutions of atmospheric relevance, along with their identities and the physical conditions of solubility in each system.

## 2 Materials and Methods

### 2.1 Sample Preparation

Solutions studied were made by mixing the chemicals listed in Table 1 with deionized water. All samples were made such that KOH was in very slight excess. The average molar ratios of KOH/DCA in the systems studied are as follows: KHC_3_H_2_O_4_ (1.008 ± 0.009), K_2_C_3_H_2_O_4_ (2.003 ± 0.006), KHC_4_H_4_O_4_ (1.02 ± 0.03), and KHC_5_H_6_O_4_ (1.001 ± 0.001). The concentrations of AS and the respective organic salt in each solution studied are given in Tables S1–S4. Throughout this paper, values reported as *a* ± *b* are mean values (a) with calculated sample standard deviation.

**Table 1:** Chemical Samples Used in This Study

Chemical name	Chemical Formula	Source	Mass fraction purity	Purification Method
Malonic Acid	C_4_H_6_O_4_	Acros	>0.99^a^	None
Succinic Acid	C_4_H_6_O_4_	Sigma-Aldrich	>0.99^a^	None
Glutaric Acid	C_5_H_8_O_4_	Acros Organics	>0.99^a^	None
Potassium Hydroxide	KOH	Fisher Scientific	0.995 ± 0.008^b^	None
Ammonium Sulfate	(NH_4_)_2_SO_4_	Fisher Scientific	1.000^a^	None
Potassium Sulfate	K_2_SO_4_	in situ^c^	^d^	None
Ammonium Potassium Sulfate	(NH_4_)_(2–*x*)_K_*x*_SO_4_	in situ^c^	^d^	None
Water	H_2_O	Municipal	≥0.9999995	R.O. + D.I.^e^

a As reported by the supplier.

b As determined by titration of KOH samples with potassium hydrogen phthalate.

c These compounds were synthesized from mixtures of commercial compounds in deionized water as described in the text.

d Our analysis could not determine the purity of the crystals formed in solution.

e Water from the municipal source was purified with a Culligan B-Series Reverse Osmosis (R.O.) system and polished with two Culligan mixed-bed deionizers (D.I.). Purity is as reported by Culligan using conductivity measurements (0.055 μS/cm).

### 2.2 Species Identification

The identity of the salt that precipitates from the solution was confirmed by a combination of X-ray crystallography (unit cell determination) and infrared spectroscopy. Unit cell determination was performed using techniques previously described in Kissinger et al.^26^ Briefly, a representative single crystal was removed from a saturated solution at room temperature and placed in the instrument for unit cell determination also at room temperature. The identity of the solid was based on a match of the unit cell parameters reported to the Cambridge Crystallographic Database (CSD) or the Inorganic Crystal Structure Database (ICSD). X-ray crystallography was utilized for all systems studied in this article, and the identity of the following salts was determined with X-ray experiments: AS,^32^ K_2_SO_4_,^33^ and (NH_4_)_(2–x)_K_*x*_SO_4_.^34,35^ For each ternary system studied, single crystals from several solutions with the same AS concentration were analyzed by X-ray crystallography.

Infrared spectroscopy was used to identify when ice formed and melted in the studied samples. Infrared spectra were acquired using a temperature-controlled, air-sealed cell, which is described elsewhere.^27^ Temperature control of the sample was achieved by resistive heating, with the cooling source being liquid nitrogen in contact with the cell. 2 μL amount of the liquid sample was placed on a ZnSe window and compressed with a second ZnSe window placed in the beam path of a Bruker Tensor 37 FTIR with a DTGS detector at 4 cm^–1^ resolution. Temperature calibration of the cell was achieved by observing the melting phase transition of several substances: HPLC-grade water, decane, octane, and acetic anhydride all supplied by Aldrich, and covering the range of 273 to 200 K.^36^

The pattern of IR spectral features in the fingerprint region was also used to determine the identity of the least soluble salt for samples for which X-ray analysis was not performed. Infrared spectra for AS and K_2_SO_4_ are well-known and available in standard databases^37^ for comparison to experimental spectra. Spectra for (NH_4_)_(2–*x*)_K_*x*_SO_4_ salts appeared to be linear additions of the spectra for AS and K_2_SO_4_ based on the amount of NH_4_^+^ and K^+^ present in the crystal (detailed in Section 3). Finally, no discontinuities or inflections were observed in the solubility data for solutions with a constant AS concentration. This observation is further indication that the same solid was the least soluble throughout a series of samples with the same AS concentration in each system.

### 2.3 Differential Scanning Calorimetry

Thermal data were obtained with a Mettler Toledo DSC1 instrument. The instrument was purged with a high-purity nitrogen gas. Temperature accuracy is estimated to be ± 0.9 K with a probability of 0.94 based on a four-point temperature calibration using indium, HPLC-grade water, anhydrous, high-purity (99%+) octane, and anhydrous high-purity heptane (99%+) from Aldrich, with the latter three stored under nitrogen. The details of the standard temperature calibration and instrument reproducibility can be found in Schubnell.^38^

Sample sizes were typically 20 ± 5 mg using 40 μL aluminum pans with sealed lids. A typical experiment consisted of initially cooling the sample from 298 K to 183 K at a rate of 10 K per minute. The sample was then held at 183 K for 5 min, and then, temperature increased 1 K per minute to 298 K or to a temperature expected to be at least five degrees above the last phase transition, while monitoring for any phase transitions that occurred during warming.

## 3 Results

### 3.1 AS/K_2_C_3_H_2_O_4_/H_2_O

Solutions that were 2:1 KOH:H_2_C_3_H_2_O_4_ in water with AS added were studied to ensure concentrations of AS in the following mass fraction series: 0.1, 0.2, 0.3, and 0.4. A complete list of concentrations studied and their solubilities is given in Table S1 and Figure 1. A typical DSC thermogram is shown in Figure S1 for a *w* = 0.3000/0.0800 AS/K_2_C_3_H_2_O_4_ sample. At a constant *w* AS, the solubility temperatures were fit to an equation as a function of *w* K_2_C_3_H_2_O_4_:

2 $$T=A_{2}x^{2}+A_{1}x+A_{0}$$

where *T* is the solubility temperature in Kelvin and *x* is the mass fraction K_2_C_3_H_2_O_4_ in the mother solution. The fit parameters *A*_*i*_ are given in Table 2.

**Table 2:** Parameters for eq 1 Used to Fit Solubility Temperatures for Each *w*AS as Given in the Table

Organic	*w*AS	*A*_2_	*A*_1_	*A*_0_
K_2_C_3_H_2_O_4_	0.1		423.92	250.04
	0.2		862.75	219.36
	0.3		1394.0	211.99
	0.4		1314.8	271.06
KHC_3_H_2_O_4_	0.02		304.44	203.88
	0.05		353.46	221.48
	0.1		410.12	224.54
	0.2		640.09	211.76
	0.3		837.51	217.58
KHC_4_H_4_O_4_	0.1	–923.50	523.80	212.41
	0.2	–4339.0	1573.6	144.25
	0.3	–2609.9	786.56	224.59
	0.4	–3567.9	710.54	243.32
KHC_5_H_6_O_4_	0.1		561.37	203.34
	0.2		905.72	180.42
	0.3		938.05	207.11
	0.4		1034.1	267.55

**Figure 1:**
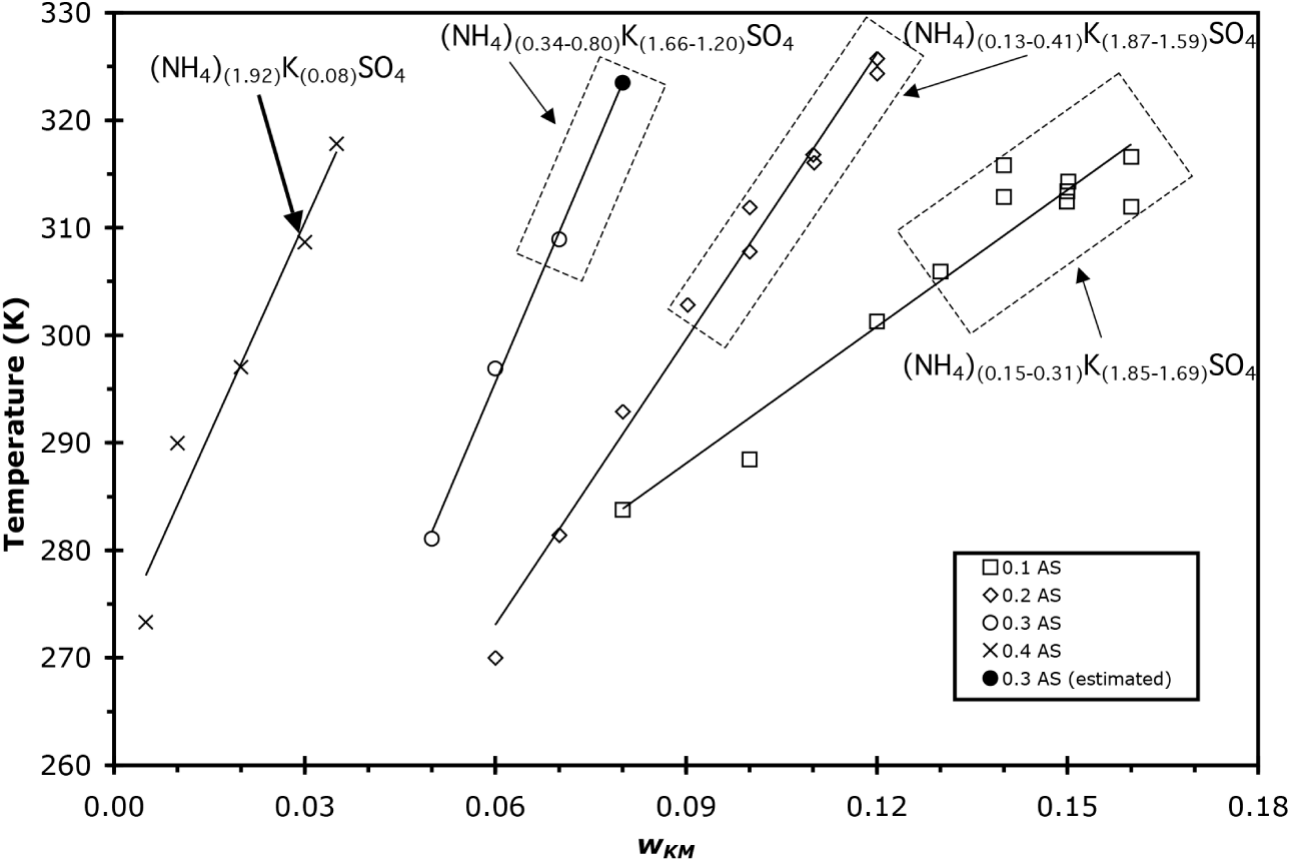
Solubilities of the least soluble compound in solutions of AS/K_2_C_3_H_2_O_4_/H_2_O from DSC and IR data where *w*_KM_ is the mass fraction of K_2_C_3_H_2_O_4_ in each solution. Concentration of AS in each solution is given in the legend in units of mass fraction (*w*). The identity of the least soluble salt, as determined by X-ray crystallography, is shown in the figure for the specific samples studied. Symbols inside dotted-line boxes were used for X-ray analysis, and crystal formulas covered a range as indicated by the respective labels. The single data point at *w* = 0.3/0.08 AS/KM (solid circle) is for a sample that did not show a clear melt in the DSC experiment but is used for X-ray crystallography. The solubility temperature for this sample is estimated from the linear fit to the other 0.3 *w* AS, as shown by the solid line for that series.

The measured unit cell parameters from crystallography experiments showed that neither K_2_C_3_H_2_O_4_ nor AS was the least soluble salt in any of these solutions. This is consistent with reported observations of the binary K_2_C_3_H_2_O_4_/H_2_O system where no salts were observed to form in DSC or IR samples.^25^ In the ternary system presented here, crystallographic data show that the compounds that formed have a mixture of NH_4_^+^/K^+^ with sulfate present in the unit cell. Crystals with the formula (NH_4_)_*x*_K_(2–*x*)_SO_4_ have been shown to have a very different composition from the composition of the mother solution,^28–30^ which agrees with the observations of this study and is discussed in more detail in Section 4. To determine the NH_4_^+^/K^+^ ratio present in the crystals studied, information from several (NH_4_)_*x*_K_(2–*x*)_SO_4_ studies in the Inorganic Crystal Structure Database (ICSD) was utilized, correlating the unit cell volume with the ammonium content of the crystal. Table S2 gives the data and references for these structures. With this data set as a basis, the unit cell volume as a function of the number of ammonium ions present in the crystal per molecular formula was plotted (with the balance of the cations being K^+^). For example, the formula (NH_4_)_0.6_K_1.4_SO_4_ would have an ammonium value of 0.6. Thus, the range of possible values for ammonium is zero for K_2_SO_4_ and 2 for AS. A plot of the data is shown in Table S2 and is shown in Figure 2 along with a linear fit to the data, with an *R* ^2^ = 0.995. Thus, the unit cell volume increases linearly with ammonium content (and decreasing K^+^ content) with slope = 30.27 Å^3^/mol NH_4_^+^ and intercept = 433.0 Å^3^. On this basis, the unit cell volumes that were determined by X-ray crystallographic analysis of our AS/K_2_C_3_H_2_O_4_/H_2_O samples were used to determine the ammonium content of the compounds that formed in each sample. The results are given in Table 3 and are shown in Figure 1 as labels identifying the compounds formed.

**Figure 2:**
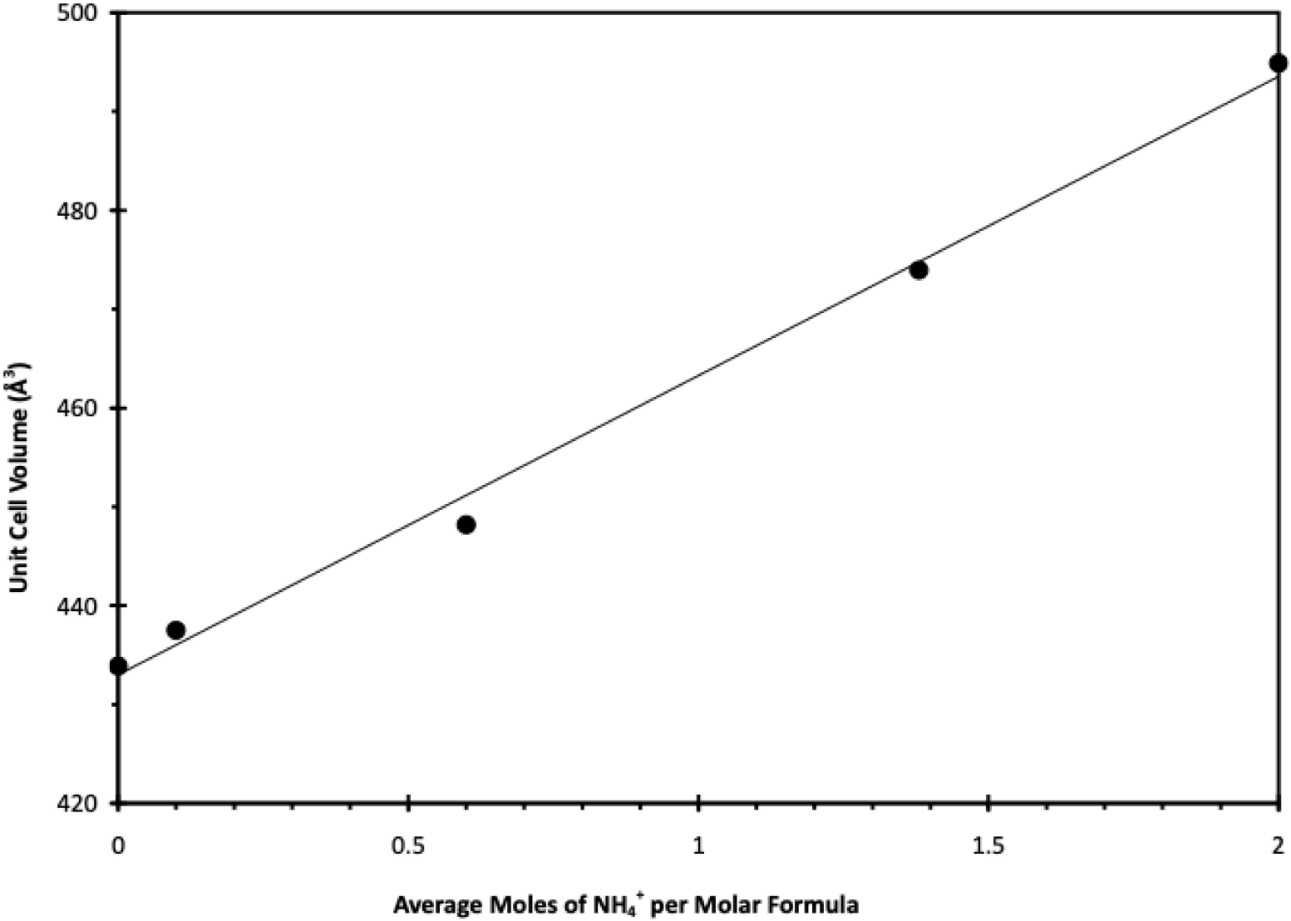
Plot of data from Table 3 correlating the unit cell volume with the average moles of ammonium ions present in the crystal per molar formula. Line is a linear fit to the literature data. Error bars for the uncertainty of the volumes are smaller than those for the symbols.

**Table 3:** Calculated Ammonium Content for Mixed NH_4_^+^/K^+^ Sulfate Crystals Formed in Ternary Solutions Where Concentration is Given in Mass Fraction (*w*), *x* is the Calculated Moles of NH_4_^+^ per Mole Formula, *y* is NH_4_^+^/(NH_4_^+^ + K^+^) in Either the Mother Solution or the Crystal on a Mole Basis, and *V* is the Unit Cell Volume

**Mother Solution**			**Crystal**			
*w*AS	*w*Organic salt	*y*	*V*/Å^3^	*x*	*y*	Average Formula
	K_2_C_3_H_2_O_4_					
0.1000	0.1300	0.45	441.0 ± 1.3	0.26 ± 0.04	0.13	(NH_4_)_0.26_K_1.74_SO_4_
0.1000	0.1400	0.43	442.5 ± 1.2	0.31 ± 0.04	0.16	(NH_4_)_0.31_K_1.69_SO_4_
0.1000	0.1500	0.42	437.6 ± 1.1	0.15 ± 0.04	0.08	(NH_4_)_0.15_K_1.85_SO_4_
0.1000	0.1600	0.40	439.4 ± 0.6	0.21 ± 0.02	0.11	(NH_4_)_0.21_K_1.79_SO_4_
0.2000	0.0902	0.70	440.9 ± 1.9	0.26 ± 0.06	0.13	(NH_4_)_0.26_K_1.74_SO_4_
0.2000	0.0999	0.68	445 ± 2	0.40 ± 0.07	0.20	(NH_4_)_0.40_K_1.60_SO_4_
0.2000	0.1100	0.66	437.0 ± 1.3	0.13 ± 0.04	0.07	(NH_4_)_0.13_K_1.87_SO_4_
0.2000	0.1200	0.64	445.3 ± 0.5	0.41 ± 0.02	0.20	(NH_4_)_0.41_K_1.59_SO_4_
0.3000	0.0700	0.82	443.2 ± 0.4	0.34 ± 0.01	0.17	(NH_4_)_0.34_K_1.66_SO_4_
0.3000	0.0800	0.80	457.1 ± 1.0	0.80 ± 0.03	0.40	(NH_4_)_0.80_K_1.20_SO_4_
0.4000	0.0300	0.93	491.1 ± 1.4	1.92 ± 0.05	0.96	(NH_4_)_1.92_K_0.08_SO_4_
	KHC_3_H_2_O_4_					
0.0200	0.3499	0.11	433.2 ± 0.07	0.01 ± 0.02	0	K_2_SO_4_
0.0501	0.2502	0.30	431.3 ± 0.8	-0.06 ± 0.03	0^a^	K_2_SO_4_
0.1000	0.2199	0.49	435.8 ± 1.0	0.09 ± 0.03	0.05	(NH_4_)_0.09_K_1.91_SO_4_
0.1999	0.1550	0.74	432.8 ± 0.8	–0.01 ± 0.03	0	K_2_SO_4_
0.3000	0.1001	0.87	452.8 ± 1.2	0.65 ± 0.04	0.33	(NH_4_)_0.65_K_1.35_SO_4_
	KHC_4_H_4_O_4_					
0.1002	0.2100	0.53	431.6 ± 0.7	–0.05 ± 0.02	0^a^	K_2_SO_4_
0.1001	0.2208	0.52	432.8 ± 0.4	–0.01 ± 0.01	0	K_2_SO_4_
0.0994	0.2280	0.51	432.7 ± 0.7	–0.01 ± 0.02	0	K_2_SO_4_
0.0998	0.2494	0.49	429.6 ± 0.1	–0.11 ± 0.00	0^a^	K_2_SO_4_
0.1995	0.1403	0.77	435.4 ± 0.2	0.08 ± 0.01	0.04	(NH_4_)_0.08_K_1.92_SO_4_
0.2004	0.1609	0.75	436.6 ± 0.7	0.12 ± 0.02	0.06	(NH_4_)_0.12_K_1.88_SO_4_
0.2000	0.1797	0.72	436.5 ± 0.3	0.12 ± 0.01	0.06	(NH_4_)_0.12_K_1.88_SO_4_
0.2999	0.0996	0.88	449.0 ± 1.0	0.53 ± 0.03	0.26	(NH_4_)_0.53_K_1.47_SO_4_
0.2994	0.1113	0.86	447.0 ± 0.6	0.46 ± 0.02	0.23	(NH_4_)_0.46_K_1.54_SO_4_
0.3000	0.1200	0.86	446.8 ± 0.6	0.46 ± 0.02	0.23	(NH_4_)_0.46_K_1.54_SO_4_
0.3002	0.1302	0.84	447.4 ± 0.7	0.48 ± 0.02	0.24	(NH_4_)_0.48_K_1.52_SO_4_
0.3994	0.0306	0.97	490.1 ± 0.8	1.89 ± 0.03	0.94	(NH_4_)_1.89_K_0.11_SO_4_
0.4009	0.0397	0.96	485.9 ± 0.4	1.75 ± 0.01	0.87	(NH_4_)_1.75_K_0.25_SO_4_
0.3995	0.0493	0.95	487.4 ± 0.9	1.80 ± 0.03	0.90	(NH_4_)_1.80_K_0.20_SO_4_
0.4003	0.0689	0.93	479.3 ± 0.5	1.53 ± 0.02	0.77	(NH_4_)_1.53_K_0.47_SO_4_
	KHC_5_H_6_O_4_					
0.1040	0.1963	0.58	441.0 ± 0.7	0.26 ± 0.02	0.13	(NH_4_)_0.26_K_1.74_SO_4_
0.1996	0.1622	0.76	448.0 ± 0.6	0.50 ± 0.02	0.25	(NH_4_)_0.50_K_1.50_SO_4_
0.3007	0.1030	0.88	489.9 ± 0.9	1.88 ± 0.03	0.94	(NH_4_)_1.88_K_0.12_SO_4_
0.2999	0.1102	0.87	467 ± 2	1.12 ± 0.07	0.56	(NH_4_)_1.12_K_0.88_SO_4_
0.4002	0.0346	0.97	496.5 ± 1.2	2.10 ± 0.04	1^b^	(NH_4_)_2_SO_4_
0.4000	0.0299	0.97	497.1 ± 0.7	2.12 ± 0.02	1^b^	(NH_4_)_2_SO_4_

a Since the calculated moles of NH_4_^+^ < 0 is physically unreal, and our crystallography colleagues interpret the X-ray diffraction results to indicate the crystal was K_2_SO_4_, we have set *y* in the crystal = 0.^39^.

b Since a calculated *x* > 2 is physically unreal, and our crystallography colleagues interpret the X-ray diffraction results to indicate the crystal was (NH_4_)_2_SO_4_, we have set *y* in the crystal = 1.^39^.

Additional evidence for the formation of mixed (NH_4_)_*x*_K_(2–*x*)_SO_4_ solids is given by comparing DSC experimental results with those reported by González-Silgo et al.,^34^ where a solid/solid phase transition accompanied by a measurable thermal signal was observed for (NH_4_)_*x*_K_(2–*x*)_SO_4_. This transition weakens (value of Δ*H* decreases), and the transition temperature decreases as the amount of NH_4_^+^ in the crystal decreases. González-Silgo et al. observed this transition for crystals with high NH_4_^+^ content (*x* > 1.66). Comparing thermal data for our sample *w* = 0.4000/0.0300 AS/K_2_C_3_H_2_O_4_, the solid/solid phase transition was observed in the thermogram (see Figure S2). The solid/solid phase transition was also observed in thermograms of other solutions with *w* = 0.4 AS (that were not used in X-ray experiments) where the transitions were also observed (Figure S2). It was also observed that the enthalpy and temperature of the transition decreased with decreasing NH_4_^+^ content, in agreement with González-Silgo et al.

As stated above, only some samples were utilized for X-ray crystallography experiments to identify the least soluble solid. For these samples, IR spectroscopy experiments were also performed, identifying the characteristic absorption bands of the least soluble solid. These characteristic absorption bands were then used in experiments on solutions that were not used for X-ray analysis to identify the least soluble solid. IR spectra and characteristic absorption bands for AS and K_2_SO_4_ solids have long been known and are found in standard references for IR spectra,^40^ as shown in Figure S3. A detailed view of the spectra shows that both salts have a strong sulfate absorption band at approximately 1110 cm^–1^. However, a characteristic absorption band for NH_4_^+^ in AS is found at 1409 cm^–1^, and a characteristic absorption band for K^+^ in K_2_SO_4_ is found at 1469 cm^–1^. Analysis of IR spectra for which X-ray experiments were also performed showed that the ratio of characteristic NH_4_^+^ and K^+^ absorption bands for a crystallized sample tracks with the ratio of NH_4_^+^/K^+^ determined to be present in the crystal from X-ray experiments. Thus, in crystallized samples, the ratio of NH_4_^+^/K^+^ absorption bands was viewed as evidence for the formation of mixed (NH_4_)_*x*_K_(2–*x*)_SO_4_ solids. Figure S4 shows the progression of the IR spectra for a solution that is *w* = 0.2001/0.0902 AS/K_2_C_3_H_2_O_4_ as it moves through a cooling and warming sequence. This sample was used for X-ray crystal analysis and was found to have an NH_4_^+^ content of 0.26 per molar formula. The ratio of IR bands predicts 0.32 for this value. The agreement seems reasonable, given that there is a variable composition of mixed crystals in a sample and that only one crystal is utilized for X-ray crystallography.

### 3.2 AS/KHC_3_H_2_O_4_/H_2_O

Solutions that were 1:1 KOH:H_2_C_3_H_2_O_4_ in water with the following mass fraction of AS were studied: 0.02, 0.05, 0.1, 0.2, and 0.3. Solutions with higher AS concentrations were attempted, but it was found that some species at these higher concentrations did not dissolve. A complete list of concentrations studied and their solubilities is given in Table S3. A typical DSC thermogram is shown in Figure S1 for a *w* = 0.3000/0.1001 AS/KHC_3_H_2_O_4_ sample. The solubility values are shown in Figure 3. At constant *w*AS, the solubility temperatures were fit to eq 1 as a function of *w* K_2_C_3_H_2_O_4_, with the fit parameters *A*_*i*_ given in Table 2.

**Figure 3:**
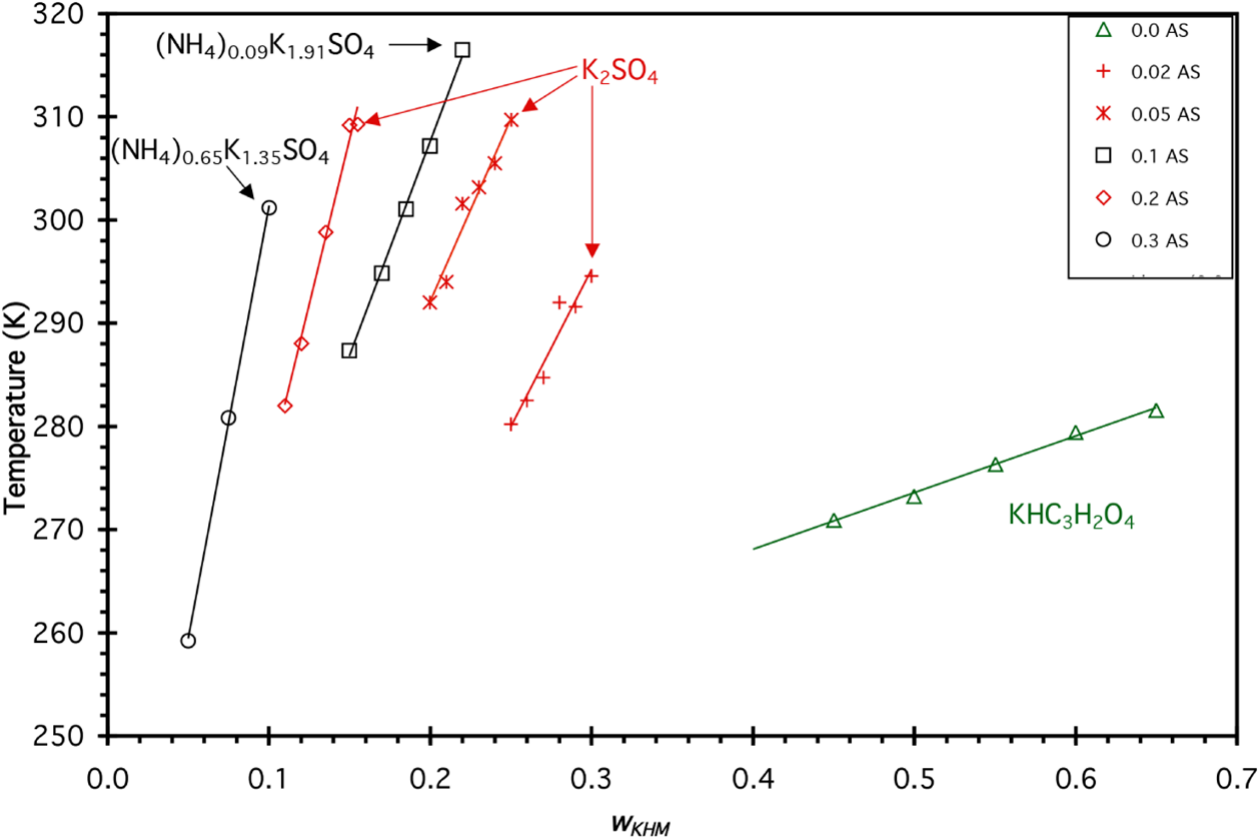
Solubilities of the least soluble compound in solutions of AS/KHC_3_H_2_O_4_/H_2_O from DSC and IR data where *w*_KHM_ is the mass fraction of KHC_3_H_2_O_4_ in each solution. Concentration of AS for each solution is given in the legend. The formula of the least soluble salt, as determined by X-ray crystallography (described in the text), is indicated by arrows. For *w* = 0.0 AS samples, the least soluble salt is KHC_3_H_2_O_4_ (green diamonds).

The measured unit cell parameters from crystallography experiments showed that neither KHC_3_H_2_O_4_ nor AS was the least soluble salt in any of these solutions. Instead, crystallographic data showed that the compounds contained a mixture of NH_4_^+^/K^+^ with sulfate present in the unit cell, or K_2_SO_4_ was formed. All of the crystals analyzed were of the formula (NH_4_)_*x*_K_(2–*x*)_SO_4_; thus, these results are similar to those found in the AS/K_2_C_3_H_2_O_4_/H_2_O system described above. The same method described in section 3.1 was used to calculate the NH_4_^+^/K^+^ ratio present in the crystals studied, and Table 3 gives the results of this analysis. Values for the ammonium content were assumed to be zero for samples where the calculated value was zero within the uncertainty of the volume measurements, thus indicating that the crystal formed was K_2_SO_4_. In one case, the calculated value for the ammonium content was −0.06 ± 0.03, but since a negative ammonium content is physically unreal, this crystal was assumed to be K_2_SO_4_. This interpretation was consistent with the X-ray data as interpreted by the collaborators who performed the crystallography experiments.^39^ The pattern seen in Figure 3 is that the NH_4_^+^ content of the crystal formed increases as *w* AS increases in the mother solution. One exception is the sample studied with *w* = 0.2 AS, where it was determined that K_2_SO_4_ was the least soluble solid in that sample via X-ray analysis. Since the sample with *w* = 0.1 AS showed some NH_4_^+^ content in the crystal from X-ray analysis, one of these two observations seems to be an anomaly. One explanation is the nature of single-crystal X-ray analysis, which only analyzes a single crystal, such that other crystals in the same mother solution may have had NH_4_^+^ in the crystal. Unfortunately, IR spectra do not aid in understanding here. With such low NH_4_^+^ content (0.09 formula moles for the *w* = 0.1 AS sample), the intensity of the NH_4_^+^ IR band is not strong enough to quantify the NH_4_^+^ content of the salt formed in solution. In general, the results for this system are similar to those of section 3.1

### 3.3 AS/KHC_4_H_4_O_4_/H_2_O

Solutions that were 1:1 KOH:H_2_C_4_H_4_O_4_ in water with the following mass fractions of AS were studied: 0.1, 0.2, 0.3, and 0.4. A complete list of concentrations studied and the liquidus temperatures of the least soluble solids formed in solution is given in Table S4. A typical DSC thermogram is shown in Figure S1 for a *w* = 0.3008/0.1176 AS/KHC_4_H_4_O_4_ sample. The solubility values are shown in Figure 4. At constant *w* AS, the solubility temperatures were fit to eq 1 as a function of *w* KHC_4_H_4_O_4_, with the fit parameters *A*_*i*_ given in Table 2. The measured unit cell parameters from crystallography experiments are given in the table and show that the least soluble solid was either K_2_SO_4_ (0.1 w AS) or (NH_4_)_*x*_K_(2–x)_SO_4_ (0.2, 0.3, and 0.4 *w* AS). It is observed that the ammonium content of the crystals formed increases with the increasing amount of AS in the mother solution, consistent with the previously described systems.

**Figure 4:**
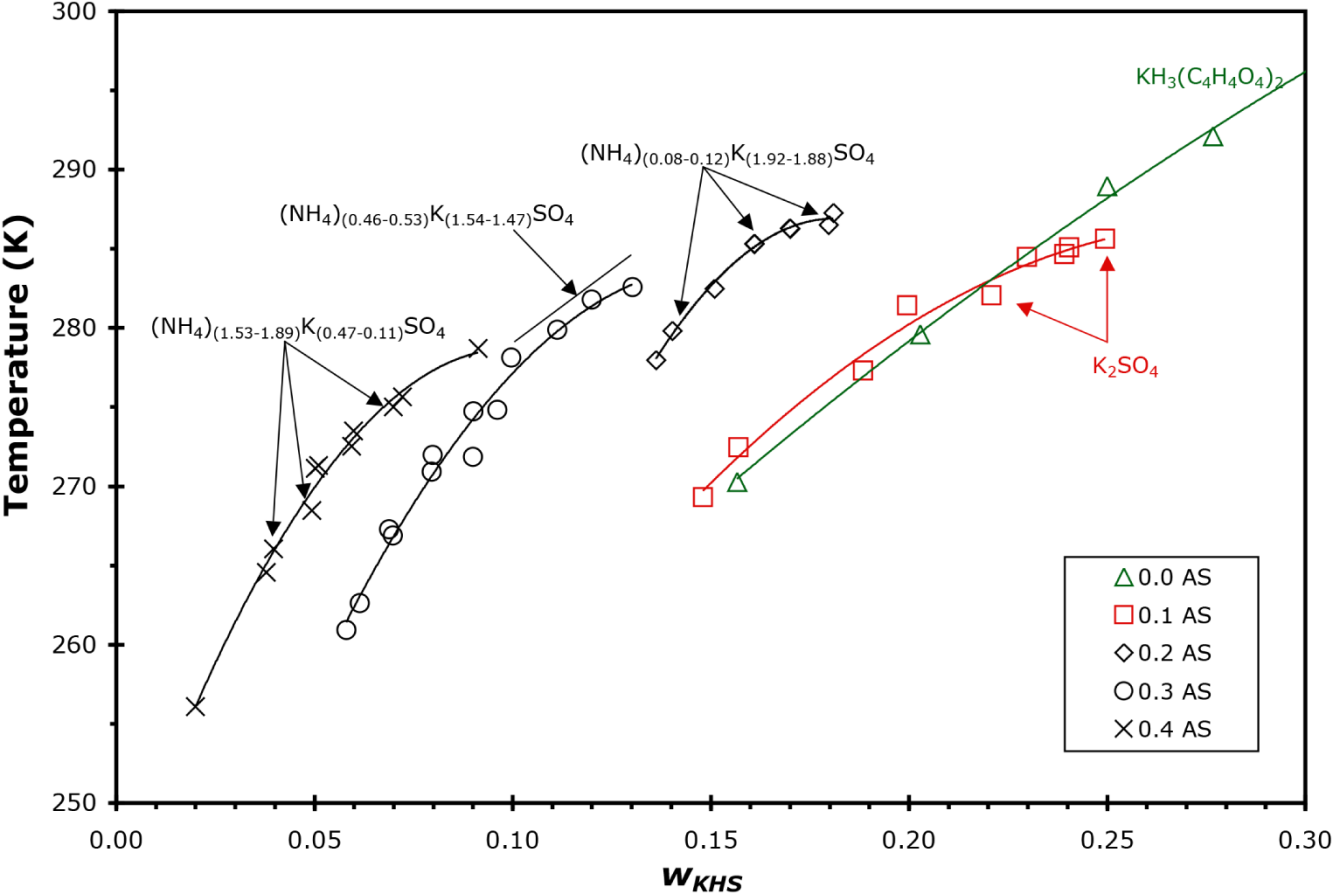
Solubilities of the least soluble compound in solutions of AS/KHC_4_H_4_O_4_/H_2_O from DSC and IR data, where *w*_KHS_ is the mass fraction of KHC_4_H_4_O_4_ in each sample. Concentration of AS of each sample is given in the legend. The identity of the least soluble salt as determined by X-ray crystallography is shown in the figure for the specific samples studied.

### 3.4 AS/KHC_5_H_6_O_4_/H_2_O

Solutions that were 1:1 KOH:H_2_C_5_H_6_O_4_ in water with the following mass fraction of AS were studied: 0.1, 0.2, 0.3, and 0.4. A complete list of concentrations studied and their solubilities is given in Table S5. A typical DSC thermogram is shown in Figure S1 for a *w* = 0.2999/0.1102 AS/KHC_5_H_6_O_4_ sample. The solubility values are shown in Figure 5. At a constant *w*AS, the solubility temperatures were fit to eq 1 as a function of *w* KHC_5_H_6_O_4_, with the fit parameters *A*_*i*_ given in Table 2. The measured unit cell parameters from crystallography experiments are given in the table and show that the least soluble solid was either (NH_4_)_*x*_K_(2–*x*)_SO_4_ (0.1, 0.2, and 0.3 *w*AS) or AS (0.4 *w*AS). As with the previous systems studied, the ammonium content in the crystals formed increased with an increasing concentration of AS in the mother solution. In fact, at the highest AS concentration (*w* = 0.4), AS is the least soluble salt. Thus, here it may be concluded that the other solids likely to form upon complete crystallization of *w* = 0.4 AS solutions would be ice and KHC_5_H_6_O_4_. It is also clear from Figure 5 that the solubility of AS is substantially less than that of the mixed solids that form at lower AS concentrations.

**Figure 5:**
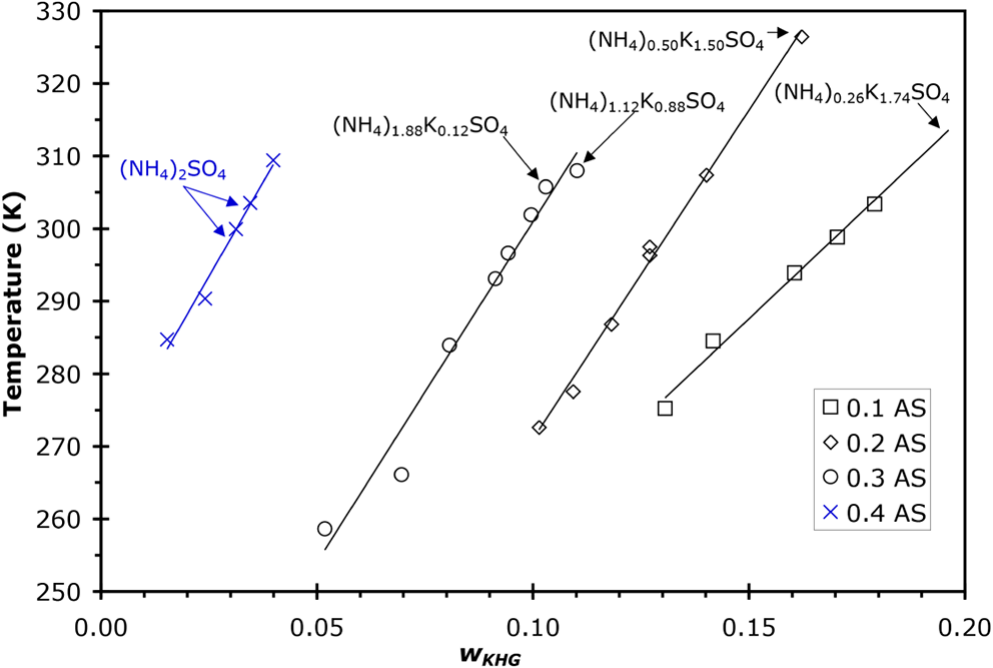
Solubilities of the least soluble compound in solutions of AS/KHC_5_H_6_O_4_/H_2_O from DSC and IR data where *w*_KHG_ is the mass fraction of KHC_5_H_6_O_4_ in each sample. Concentration of AS of each sample is given in the legend. The identity of the least soluble salt as determined by X-ray crystallography is shown in the figure for the specific samples studied.

### 3.5 AS/K_2_C_4_H_4_O_4_/H_2_O and AS/K_2_C_5_H_6_O_4_/H_2_O

Many solutions over a significant concentration range in these two systems were made; however, there were no solutions where all solids dissolved below 50 °C. Above that temperature, there is significant water evaporation, and thus, solute concentrations cannot be reliably determined.

## 4 Discussion and Atmospheric Implications

In all of the ternary systems studied, it was determined that many formed a mixed (NH_4_)_*x*_K_(2–*x*)_SO_4_ compound as the least soluble solid. As stated in Section 3.1, compounds of formula (NH_4_)_*x*_K_(2–*x*)_SO_4_ have been known to form from aqueous mixtures of K_2_SO_4_ and AS as a continuous solid solution,^28–30^ and solubilities of compounds with various compositions are found in the compilation of Linke.^31^ To compare the amount of NH_4_^+^ present in the crystals formed as a function of NH_4_^+^ present in the mother solution, a parameter that takes into account the ratio of ammonium to potassium ions in each phase is needed. For this purpose, the ratio NH_4_^+^/(NH_4_^+^ + K^+^) on a mole basis is used and given the symbol *y*. Thus, the value of this parameter is zero for a K_2_SO_4_ crystal and 1 for an AS crystal. If the value of *y* in the mother solution equals the value of *y* in the crystal formed, then a plot of these two parameters will yield a straight line from coordinates 0,0 to 1,1. The value of *y* for the mother solution versus the crystal formed from that solution (as listed in Table 3) is plotted in Figure 6 along with literature data at 298 K. As can be seen, there is a strong correlation between the different data sets, and the general shape of the trends follows that given by Calvo and Simons^30^ in their study of the aqueous AS/K_2_SO_4_ system. Their conclusion was that this system is highly nonideal, and the relationship between the fraction of NH_4_^+^ in precipitated crystals and in the mother solution cannot be represented by a polynomial equation except over short concentration ranges. What is apparent is that the ammonium content of the crystal is much lower than in the mother solution for all solutions except those with very high NH_4_^+^ content. For *y* greater than about 0.95, the value of *y* in solution and crystal phases becomes equal. What is also readily observed is that the literature data, where no organic species are present, are coincident with the data from this study, which has a wide range of organic content (both concentration in the mother solution and the identity of the organic present). Thus, it is reasonable to conclude that the organic species have little to no effect on the identity of the least soluble salt, and rather the salt formed is a function of the ammonium and potassium ion content in the mother solution. This conclusion may be highly predictive for the identity of the least soluble solid in atmospheric aerosols with K^+^ and AS content. Here, the key factor will likely be knowing the concentration of potassium, ammonium, and sulfate ions in these solutions.

**Figure 6:**
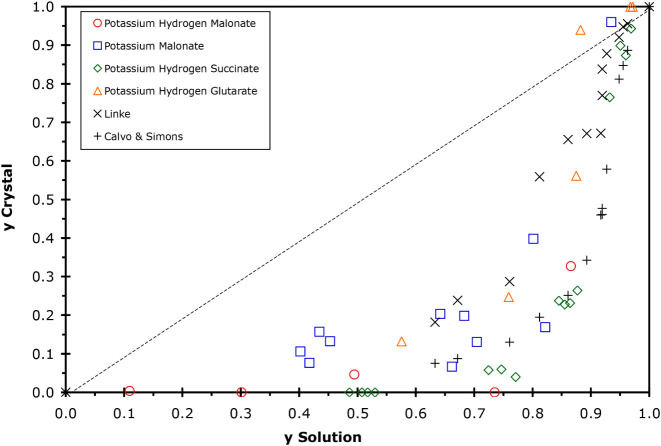
Correlation between the fraction of NH_4_^+^ in the mother solution and the fraction of NH_4_^+^ in the crystal formed from solutions of AS + KOH + organic acid, as given in the legend. The variable “*y*” is the fraction of NH_4_^+^ present out of the total of (NH_4_^+^ + K^+^) present in the respective phases on a mole basis. Values for crystals formed in aqueous solutions of AS/K_2_SO_4_ at 298 K found in Linke^31^ and those from Calvo and Simons^30^ are also shown. Dashed lines indicate a 1:1 (ideal) ratio of NH_4_^+^ in the mother solution and NH_4_^+^ in the crystals formed.

Another question is whether the identity of the organic species present has an effect on the *solubility* of the mixed (NH_4_)_*x*_K_(2–*x*)_SO_4_ salt that forms. For the solutions studied here, only solutions with *w*AS = 0.3 were observed to have a mixed salt as the least soluble compound for *all samples studied* with X-ray crystallography within an AS concentration series (for other AS concentration series, either AS or K_2_SO_4_ formed in some solutions). To make a comparison based on moles of organic content, the mole fraction of the organic content was plotted vs. solubility temperature for all samples with *w*AS = 0.3, as shown in Figure 7. It is seen that the identity of the organic species present in the mother solution does have an impact on the solubility of the least soluble salt. The (NH_4_)_*x*_K_(2–*x*)_SO_4_ compounds are most soluble in potassium hydrogen succinate solutions and least soluble in potassium malonate solutions. However, at low temperatures, the solubility converges for solutions with KHC_4_H_4_O_4_, KHC_3_H_2_O_4_, and KHC_5_H_6_O_4_ present. Thus, at temperatures above about 260 K, the organic species present does affect the solubility of the mixed compounds for the single potassium salt solutions. In addition, the mixed compounds are least soluble in solutions containing potassium and malonate in a 2:1 ratio (labeled as K_2_C_3_H_2_O_4_ in Figure 7) at all temperatures studied here.

**Figure 7:**
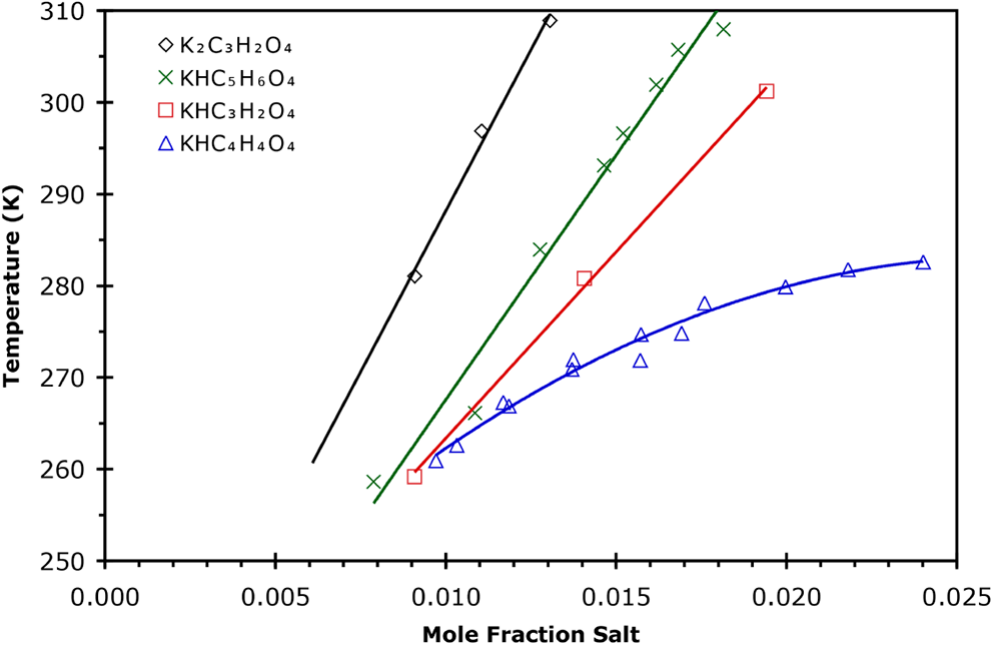
Plot of the solubility of (NH_4_)_*x*_K_(2–*x*)_SO_4_ as a function of the mole fraction of organic salt for solutions with *w*AS = 0.3. The symbol for each organic species is given in the legend.

The least soluble solids in solutions of AS with sodium salts of dicarboxylic acids have been previously reported^26,27^ and are summarized in Table 4. These observations are compared to those of the least soluble solids found in the systems studied here. It is readily apparent that there are significant differences between the solutions with Na ions and those with K ions in the trends for the least soluble solid. In general, the potassium organic salts were not observed as the least soluble salt in those solutions, whereas in solutions with sodium ions present, the organic sodium salts were the least soluble solid under various conditions. In the AS/NaHC_3_H_2_O_4_ system at low AS concentrations (*w* = 0.1), NaSO_4_·10H_2_O was found to be the least soluble salt. At higher AS concentrations (*w* = 0.2 and 0.3), NaNH_4_SO_4_·H_2_O (lecontite) was found to be the least soluble salt. In that system, the solubility temperature varied only weakly with the concentration of NaHC_3_H_2_O_4_. In stark contrast to this, the solubility of the mixed (NH_4_)_*x*_K_(2–*x*)_SO_4_ compounds in the AS/KHC_3_H_2_O_4_/H_2_O system is strongly dependent on KHC_3_H_2_O_4_ concentration, as seen in Figure 5. It is observed that these mixed (NH_4_)_*x*_K_(2–*x*)_SO_4_ compounds are much less soluble in water than lecontite for similar organic salt concentrations.

**Table 4:** Summary of the Least Soluble Solids in Solutions Studied Here (K^+^ Ions Present) and in Previous Studies (Na^+^ Ions Present)^26,27^ in the “Na Solutions” Column, the Mass Fractions (*w*) Listed are for as Content^a^

Organic anion	Na solutions	K solutions
C_3_H_2_O_4_^2–^	*w* = 0.1, 0.2: Na_2_SO_4_·10H_2_O & Lecontite	(NH_4_)_*x*_K_(2–x)_SO_4_
	*w* = 0.3: Lecontite	
HC_3_H_2_O_4_^–^	*w* = 0.1: Na_2_SO_4_·10H_2_O	K_2_SO_4_ & (NH_4_)_*x*_K_(2–x)_SO_4_
	*w* = 0.2, 0.3: Lecontite	
HC_4_H_4_O_4_^–^	*w* = 0.1: NaHC_4_H_4_O_4_ & Lecontite	K_2_SO_4_ & (NH_4_)_*x*_K_(2–x)_SO_4_
	*w* = 0.2: Lecontite	
	*w* = 0.3: AS & Lecontite	
	*w* = 0.4: AS	
HC_5_H_6_O_4_^–^	*w* = 0.1: NaHC_5_H_6_O_4_·2H_2_O & Lecontite	(NH_4_)_*x*_K_(2–x)_SO_4_ & AS
	*w* = 0.2: Lecontite	
	*w* = 0.3, 0.4: NH_4_HC_5_H_6_O_4_	

a In each instance where two compounds are listed, the one with a higher ammonium content is observed at a higher ammonium concentration in the mother solution .

Similar differences are seen between the potassium malonate and sodium malonate systems in the presence of AS. The least soluble solids in solutions with sodium ions were found to be either Na_2_SO_4_·10H_2_O or lecontite.^26^ For solutions with potassium ions present, the least soluble solids were mixed with (NH_4_)_*x*_K_(2–*x*)_SO_4_ compounds. However, as in the AS/KHC_3_H_2_O_4_/H_2_O system, these mixed (NH_4_)_*x*_K_(2–*x*)_SO_4_ compounds are less soluble than either lecontite or Na_2_SO_4_·10H_2_O for similar organic salt concentrations. Also, the mixed (NH_4_)_*x*_K_(2–*x*)_SO_4_ compound solubilities had a modest temperature dependence, whereas lecontite solubility displayed a strong temperature dependence in previous studies, which appeared independent of AS concentration.^26^ This latter effect was not the case for the mixed (NH_4_)_*x*_K_(2–*x*)_SO_4_ compounds, as their solubility was strongly dependent on AS concentration.

For solutions containing sodium ions with hydrogen succinate and AS, lecontite was found to be the least soluble solid at NH_4_^+^/Na^+^ ratios greater than 1. At ratios less than 1, NaHC_4_H_4_O_4_ was found to be the least soluble. However, at very high ratios, the least soluble solid was found to be AS.^27^ In contrast, solutions containing potassium ions with hydrogen succinate and AS in this study were not observed to have an organic salt as the least soluble solid even at very low ammonium content; rather, K_2_SO_4_ was the least soluble. At moderate to higher ammonium content, (NH_4_)_*x*_K_(2–*x*)_SO_4_ compounds were the least soluble. Similar observations can be made for the solutions of sodium with hydrogen glutarate and AS studied previously.^27^ The sodium salt of the organic species is the least soluble at NH_4_^+^/Na^+^ ratios less than 1: NaHC_5_H_6_O_4_·2H_2_O. At moderate ratios, lecontite is least soluble, and at high ratios, NH_4_HC_5_H_6_O_4_ is least soluble. Here. again, compared to solutions with potassium present instead of sodium, (NH_4_)_*x*_K_(2–*x*)_SO_4_ compounds were the least soluble over a wide composition range, with AS being the least soluble at the highest ammonium concentrations.

A rationale for why (NH_4_)_*x*_K_(2–*x*)_SO_4_ compounds form over a wider concentration range of ammonium content in solutions containing potassium ions vs. lecontite in the sodium-containing solutions is that (NH_4_)_*x*_K_(2–*x*)_SO_4_ compounds have a wide range of possible ammonium composition. In contrast, lecontite has a fixed ammonium-to-sodium ratio: NaNH_4_SO_4_·H_2_O. Thus, a ratio of NH_4_^+^/Na^+^ of around 1 or somewhat greater favors its formation; whereas, at very high ratios, AS is favored. Therefore, the presence of potassium ions could have a significant salting-out effect on ammonium and sulfate in aqueous solutions over a wide concentration range of ions by forming mixed (NH_4_)_*x*_K_(2–*x*)_SO_4_ compounds preferentially to lecontite formation in the presence of sodium ions. Therefore, knowledge of the potassium ion content may be more important than the sodium ion content for determining the physical conditions under which solids will precipitate in atmospheric aerosols.

## 5 Conclusions

The solubility and identity of the least soluble compound in several ternary systems important for understanding composition in atmospheric aerosols have been studied: aqueous dicarboxylates with potassium ions and AS. In the range of systems studied, varying both organic species and concentrations, the organic fraction appeared to have no bearing on the identity of the least soluble solid, as they were all inorganic. In solutions where the mixed salt (NH_4_)_(2–x)_K_*x*_SO_4_ was identified as the least soluble, the composition of the salt formed follows the trend previously observed in literature studies of the K_2_SO_4_/AS/H_2_O system.^30,31^ In solutions in which the mixed salt formed, the organic fraction appears to have an impact on the solubility of the respective mixed solids that form. In comparison to the analogous ternary systems involving sodium ions rather than potassium ions, the potassium salts were generally less soluble than the sodium salts previously identified.^26,27^

## Supplementary Material



## Notes

Solubility data for all systems; literature unit cell data used to determine values of *x* and *y*; sample DSC thermograms; IR spectra for mixed solids (PDF)
