# Supplementary material for: Solubilities in Aqueous Solutions of Ammonium Sulfate and Potassium Salts of Malonic, Succinic, or Glutaric Acid
Source: J Phys Chem A. 2025 Aug 19;129(34):7917–26. doi: 10.1021/acs.jpca.5c05396 (PMC12400424; doi:10.1021/acs.jpca.5c05396)
Supplement: Supplementary file 1 [file jp5c05396_si_001.pdf]

Solubilities in Aqueous Solutions of Ammonium Sulfate and Potassium Salts of Malonic, Succinic, or Glutaric Acid

*Keith D. Beyer**, *Karl T. Taylor, Samuel R. Rendler, Julia L. Roskam*

Department of Chemistry & Biochemistry, University of Wisconsin-La Crosse, La Crosse, WI, 54601, USA

**Correspondence to*: Keith D. Beyer (kbeyer@uwlax.edu)

Supporting Information

Table S1. Solubility of the least soluble solid in the $(NH_4)_2SO_4/K_2C_3H_2O_4/H_2O$ system. First two columns give the mother solution content in mass fraction (w), T_l is the solubility temperature in Kelvin.

w $(NH_4)_2SO_4$	w $K_2C_3H_2O_4$	T_l/K	*Crystal Formula*[a]
0.1000	0.0800	283.75	
0.1000	0.1000	288.44	
0.1000	0.1200	301.28	
0.1000	0.1300	305.92	$(NH_4)_{0.26}K_{1.74}SO_4$
0.1000	0.1400	312.85	$(NH_4)_{0.31}K_{1.69}SO_4$
0.1000	0.1400	315.82	$(NH_4)_{0.31}K_{1.69}SO_4$
0.0998	0.1499	312.43	
0.0998	0.1499	320.44	
0.1000	0.1501	314.32	
0.1000	0.1500	313.38	$(NH_4)_{0.15}K_{1.85}SO_4$
0.1000	0.1600	311.97	$(NH_4)_{0.21}K_{1.79}SO_4$
0.1000	0.1600	316.60	$(NH_4)_{0.21}K_{1.79}SO_4$
0.2001	0.0600	270.00	
0.2000	0.0700	281.41	
0.2001	0.0800	292.91	
0.2000	0.0902	302.84	$(NH_4)_{0.26}K_{1.74}SO_4$
0.2000	0.0999	317.96	$(NH_4)_{0.40}K_{1.60}SO_4$
0.2000	0.1000	307.80	
0.2000	0.1000	311.91	
0.1999	0.1101	316.06	
0.2000	0.1100	316.83	$(NH_4)_{0.13}K_{1.87}SO_4$
0.1999	0.1200	324.37	
0.2000	0.1200	325.75	$(NH_4)_{0.41}K_{1.59}SO_4$
0.3000	0.0500	281.06	
0.3000	0.0600	296.89	
0.3000	0.0700	308.94	$(NH_4)_{0.34}K_{1.66}SO_4$
0.3000	0.0800	[b]	$(NH_4)_{0.80}K_{1.20}SO$
0.4000	0.0050	273.32	
0.4000	0.0100	289.95	
0.4000	0.0200	297.02	
0.4000	0.0300	308.68	$(NH_4)_{1.92}K_{0.08}SO_4$
0.4000	0.0350	317.81	

[a]Average crystal formula from X-ray crystallography as given in Table 4.
[b]A clear final solubility temperature was not discernable in DSC and/or IR experiments; however, a crystal from this solution was utilized for X-ray crystallography and so is included here.

Table S2. Literature data used to determine ammonium and potassium content of crystals formed in the ternary systems in this study where x is the number of moles of NH_4^+ per molar formula, y is $NH_4^+/(NH_4^+ + K^+)$ in the crystal on a mole basis, and V is the unit cell volume.

Formula	*x*	*y*	*V*/Å^3	Reference
K_2SO_4	0	0	433.9±0.3	28
$(NH_4)_{(0.10)}(K)_{(1.90)}(SO_4)$	0.10	0.050	437.5±0.2	29
$(NH_4)_{(0.60)}(K)_{(1.40)}(SO_4)$	0.60	0.300	448.2±0.1	30
$(NH_4)_{(1.38)}(K)_{(0.62)}(SO_4)$	1.38	0.690	474.0±0.1	30
$(NH_4)_2SO_4$	2	1	494.89±0.03	27

Table S3. Solubility of the least soluble solid in the $(NH_4)_2SO_4/KHC_3H_2O_4/H_2O$ system. First two columns give the mother solution content in mass fraction (w), T_l is the solubility temperature in Kelvin.

w $(NH_4)_2SO_4$	w $KHC_3H_2O_4$	T_l/K	*Crystal Formula*[a]
0.0200	0.2500	280.2	
0.0201	0.2600	282.5	
0.0200	0.2701	284.8	
0.0200	0.2800	292	
0.0200	0.2900	291.6	
0.0200	0.3000	294.6	
0.0200	0.3499	[b]	K_2SO_4
0.0500	0.1997	292	
0.0500	0.2100	294	
0.0500	0.2200	301.6	
0.0500	0.2300	303.2	
0.0500	0.2400	305.5	
0.0501	0.2502	309.7	K_2SO_4
0.0999	0.1497	287.4	
0.1000	0.1699	294.9	
0.1000	0.1850	301.1	
0.0999	0.1998	307.2	
0.1000	0.2199	316.5	$(NH_4)_{0.09}K_{1.91}SO_4$
0.1999	0.1099	282	
0.2000	0.1200	288.1	
0.2000	0.1350	298.9	
0.1997	0.1498	309.2	
0.1999	0.1550	309.3	K_2SO_4
0.3000	0.0500	259.2	
0.2999	0.0750	280.8	
0.3000	0.1001	301.2	$(NH_4)_{0.65}K_{1.35}SO_4$

[a]Average crystal formula from X-ray crystallography as given in Table 4.
[b]A clear final solubility temperature was not discernable in DSC and/or IR experiments; however, a crystal from this solution was utilized for X-ray crystallography and so is included here.

Table S4. Solubility of the least soluble solid in the $(NH_4)_2SO_4/KHC_4H_4O_4/H_2O$ system. First two columns give the mother solution content in mass fraction (w), T_l is the solubility temperature in Kelvin.

w AS	w $KHC_4H_4O_4$	T_l/K	*Crystal Formula*[a]
0.1002	0.1480	269.33	
0.1003	0.1570	272.47	
0.1001	0.1884	277.32	
0.1000	0.1995	281.44	
0.1002	0.2100	[b]	K_2SO_4
0.1001	0.2208	282.07	K_2SO_4
0.1000	0.2298	284.48	
0.1000	0.2280	[b]	K_2SO_4
0.0997	0.2392	284.68	
0.0996	0.2404	285.10	
0.0998	0.2494	285.66	K_2SO_4
0.1993	0.1361	277.97	
0.1995	0.1403	279.82	$(NH_4)_{0.08}K_{1.92}SO_4$
0.2004	0.1509	282.50	
0.2002	0.1609	285.34	
0.2004	0.1609	285.30	$(NH_4)_{0.12}K_{1.88}SO_4$
0.2003	0.1699	286.30	
0.2000	0.1700	286.27	
0.2000	0.1797	286.52	$(NH_4)_{0.12}K_{1.88}SO_4$
0.1997	0.1809	287.27	
0.3008	0.0580	260.94	
0.3004	0.0614	262.62	
0.3009	0.0688	267.3	
0.2998	0.0698	266.90	
0.3002	0.0796	270.92	
0.3004	0.0798	271.99	
0.3001	0.0900	271.88	
0.3004	0.0901	274.73	
0.3008	0.0961	274.83	
0.2999	0.0996	278.14	$(NH_4)_{0.53}K_{1.47}SO_4$
0.2994	0.1113	279.89	$(NH_4)_{0.46}K_{1.54}SO_4$
0.3000	0.1200	281.80	$(NH_4)_{0.46}K_{1.54}SO_4$
0.3002	0.1302	282.59	$(NH_4)_{0.48}K_{1.52}SO_4$
0.4001	0.0200	256.08	
0.3994	0.0306	[b]	$(NH_4)_{1.89}K_{0.11}SO_4$
0.4008	0.0378	264.58	
0.4009	0.0397	266.04	$(NH_4)_{1.75}K_{0.25}SO_4$
0.3995	0.0493	268.47	$(NH_4)_{1.80}K_{0.20}SO_4$
0.4001	0.0502	271.15	

0.3997	0.0510	271.32	
0.4004	0.0594	272.52	
0.3998	0.0599	273.50	
0.4003	0.0689	[b]	$(NH_4)_{1.53}K_{0.47}SO_4$
0.4006	0.0699	275.00	
0.3992	0.0722	275.66	
0.3990	0.0913	278.72	

[a]Average crystal formula from X-ray crystallography as given in Table 4.
[b]A clear final solubility temperature was not discernable in DSC and/or IR experiments; however, a crystal from this solution was utilized for X-ray crystallography and so is included here.

Table S5. Solubility of the least soluble solid in the $(NH_4)_2SO_4/KHC_5H_6O_4/H_2O$ system. First two columns give the mother solution content in mass fraction (*w*), T_l is the solubility temperature in Kelvin.

w AS	*w* $KHC_4H_4O_4$	T_l/K	*Crystal Formula*[a]
0.1000	0.1306	275.25	
0.0999	0.1417	284.52	
0.1001	0.1605	293.9	
0.0997	0.1705	298.86	
0.1000	0.1791	303.38	
0.1004	0.1963	[b]	$(NH_4)_{(0.26)}K_{(1.74)}SO_4$
0.1996	0.1014	272.61	
0.2000	0.1093	277.56	
0.2005	0.1182	286.85	
0.2007	0.1270	296.33	
0.2007	0.1270	297.49	
0.2000	0.1401	307.4	
0.1996	0.1622	326.46	$(NH_4)_{(0.50)}K_{(1.50)}SO_4$
0.2992	0.0518	258.62	
0.3002	0.0696	266.11	
0.2999	0.0807	283.93	
0.2995	0.0913	293.13	
0.3003	0.0943	296.6	
0.3002	0.0996	301.92	
0.3007	0.1030	305.74	$(NH_4)_{(1.88)}K_{(0.12)}SO_4$
0.2999	0.1102	307.97	$(NH_4)_{(1.12)}K_{(0.88)}SO_4$
0.4000	0.0153	284.7	
0.4000	0.0241	290.33	
0.4000	0.0299	[b]	$(NH_4)_2SO_4$
0.3994	0.0313	299.92	
0.4002	0.0346	303.54	$(NH_4)_2SO_4$
0.4000	0.0399	309.41	

[a]Average crystal formula from X-ray crystallography as given in Table 4.
[b]A clear final solubility temperature was not discernable in DSC and/or IR experiments; however, a crystal from this solution was utilized for X-ray crystallography and so is included here.

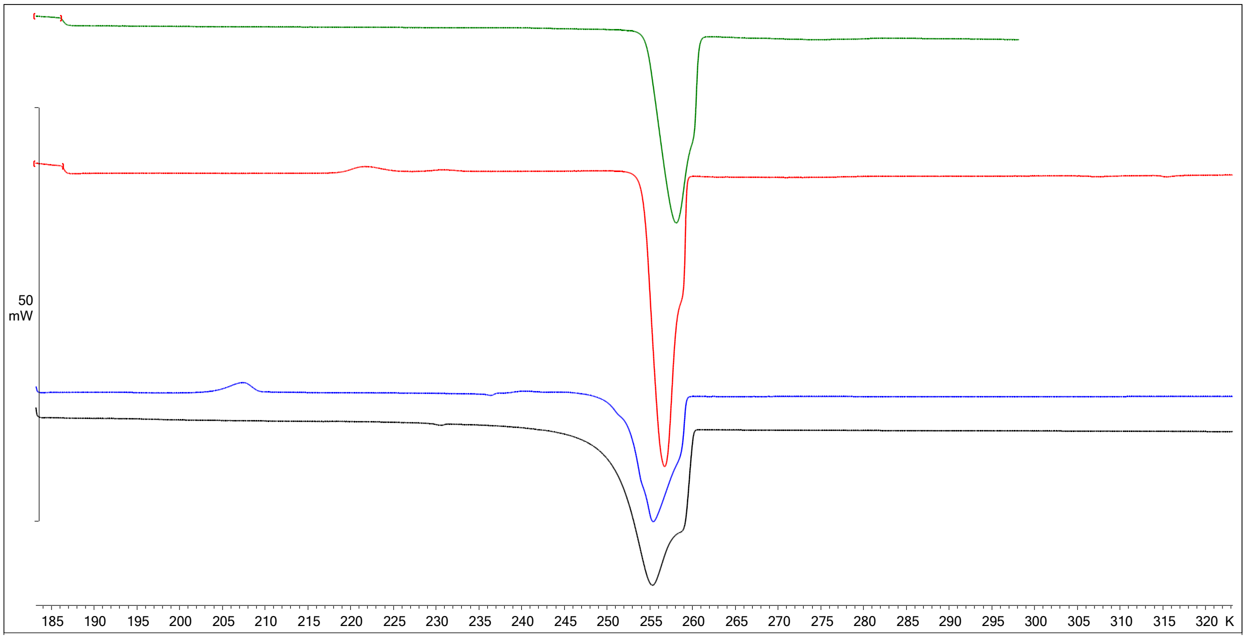

Figure S1. Typical DSC thermograms (warming segment) for the systems studied in this paper. Thermograms are: black, w = 0.3000/0.0800 AS/$K_2C_3H_2O_4$; blue, w = 0.3000/0.1001 AS/$KHC_3H_2O_4$; red, w = 0.2999/0.1102 AS/$KHC_5H_6O_4$; green, w = 0.3008/0.1176 AS/$KHC_4H_4O_4$.

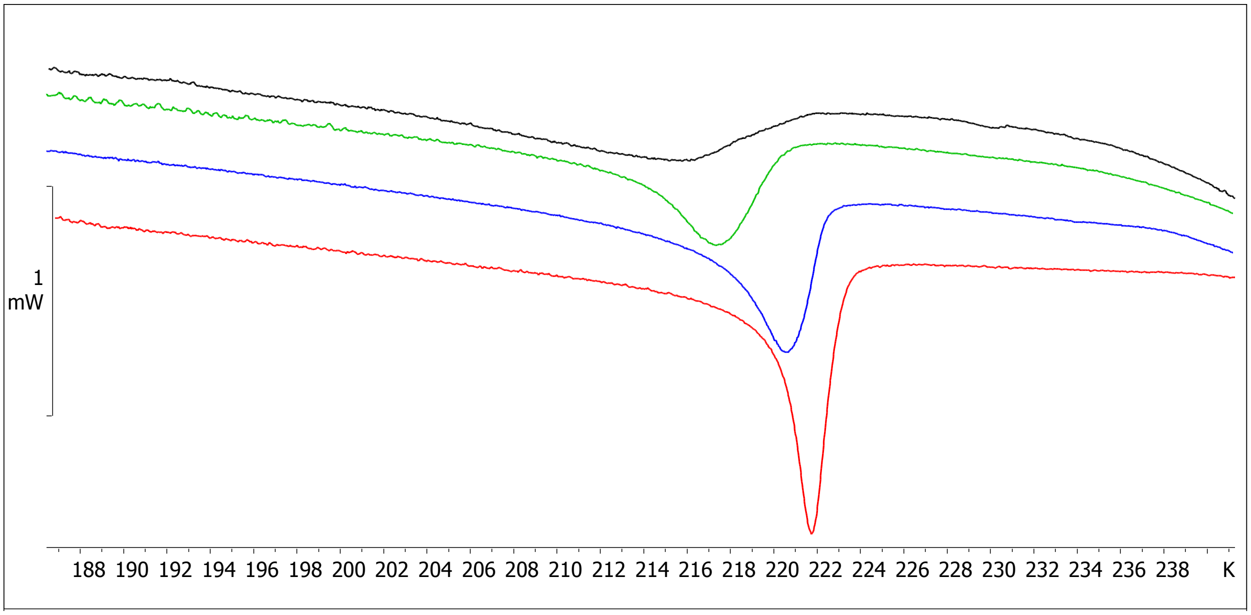

Figure S2. DSC thermograms of w = 0.4000 $(NH_4)_2SO_4$ in $(NH_4)_2SO_4/K_2C_3H_2/H_2O$ samples showing a solid/solid phase transition (with maximum value at 215 – 222 K) due to mixed $(NH_4)_xK_{(2-x)}SO_4$ solids. Colors are the following w $K_2C_3H_2O_4$: red 0.0050; blue 0.0100; green 0.0200; black 0.0300. It is observed that the ΔH and temperature of the transition decreases with increasing amount of potassium present in agreement with the observation of Gonzalez-Sligo, et al. [27].

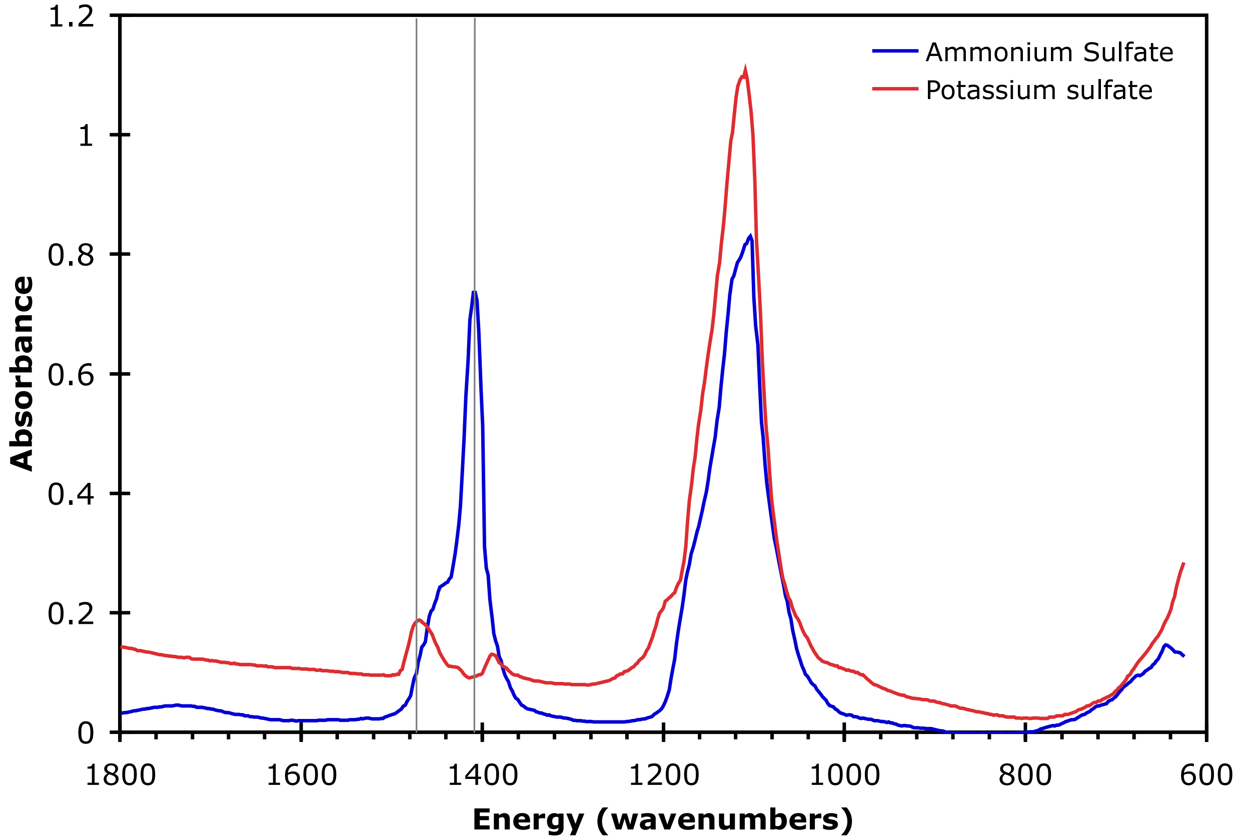

Figure S3. Fingerprint region of the infrared spectra of ammonium sulfate and potassium sulfate both in nujol mull as reported by NIST. Concentration of each species in the sample is not given by NIST, but observing the sulfate band centered at approximately 1110 cm^{-1} and comparing the baseline for each salt at 1275 cm^{-1} shows a small offset in absorbance values of 0.063. Absorbance due to NH_4^+ in ammonium sulfate is a maximum at1409 cm^{-1}, and the absorbance due to K^+ in potassium sulfate is a maximum at 1469 cm^{-1} (values marked by gray vertical lines).

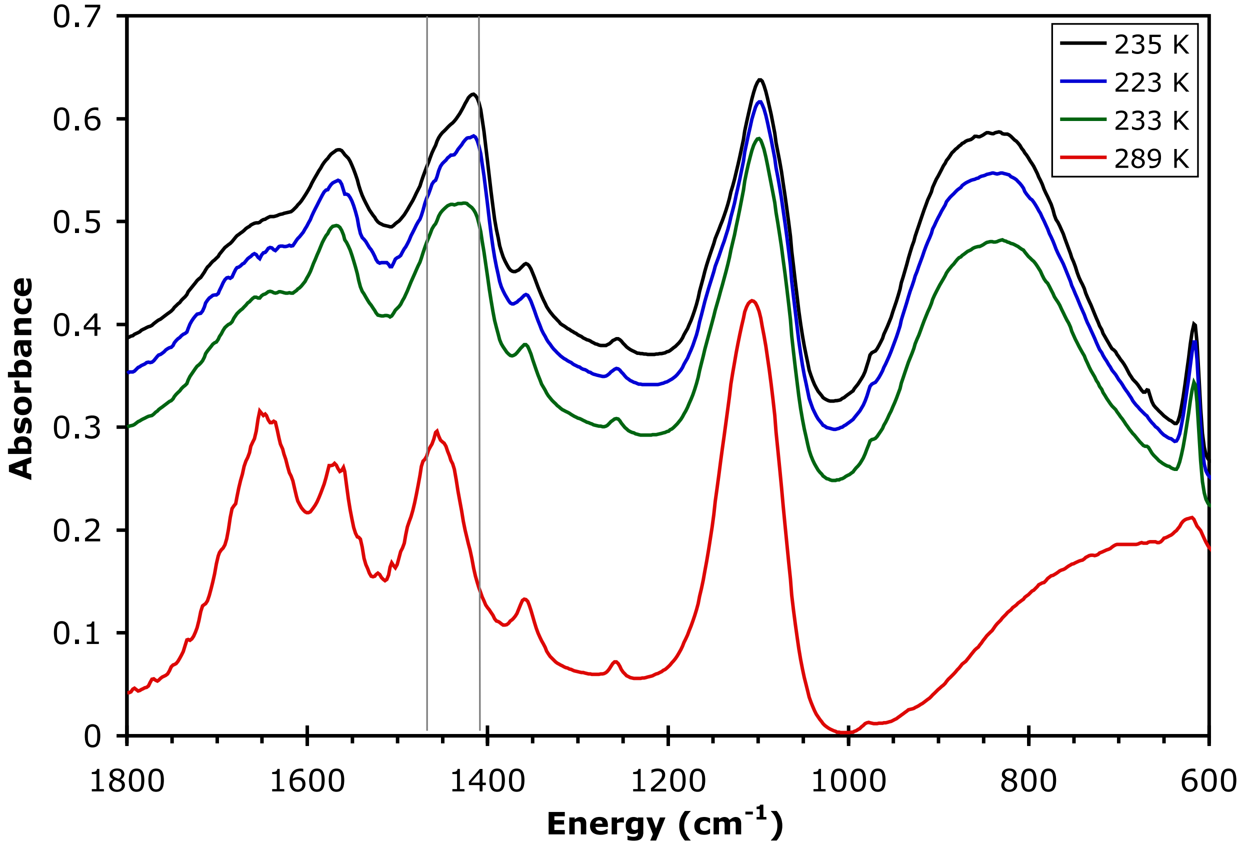

Figure S4. Portion of the infrared spectra for a solution that is *w* = 0.2001 $(NH_4)_2SO_4$/0.0902 $K_2C_3H_2O_4$, showing several points in the cooling/warming process with temperatures given in the legend. Red spectrum shows completely liquid sample. Green spectrum was recorded after ice froze in the cooling segment as indicated by the large shift in the OH band from 700 to 820 cm^{-1}. Blue spectrum indicates the remainder of the sample crystallized as the sample was further cooled. Black spectrum is in the warming segment and was taken just before melting of the sample began. Vertical gray lines are set at 1469 cm^{-1} (indicating K^+ content) and 1409 cm^{-1} (indicating NH_4^+ content). The ratio of the absorbances at these two values is indicative of the ratio of NH_4^+/K^+ in solid $(NH_4)_xK_{(2-x)}SO_4$ formed in the sample. The ratio for the black spectrum is 1.34 predicting x = 0.32 in the crystal. X-ray crystallography results indicate x = 0.26 for a single crystal from this solution.
